# The Effect of Short- and Long-Term Cryopreservation on Chicken Primordial Germ Cells

**DOI:** 10.3390/genes15050624

**Published:** 2024-05-14

**Authors:** Mariam Ibrahim, Ewa Grochowska, Bence Lázár, Eszter Várkonyi, Marek Bednarczyk, Katarzyna Stadnicka

**Affiliations:** 1 Department of Animal Biotechnology and Genetics, Bydgoszcz University of Science and Technology, Mazowiecka 28, 85-084 Bydgoszcz, Poland; 2 PBS Doctoral School, Bydgoszcz University of Science and Technology, Aleje Prof. S. Kaliskiego 7, 85-796 Bydgoszcz, Poland; 3 National Centre for Biodiversity and Gene Conservation, Institute for Farm Animal Gene Conservation, Isaszegi Street 200, 2100 Godollo, Hungary; 4 Institute of Genetics and Biotechnology, Hungarian University of Agriculture and Life Sciences, Szent-Gyorgyi Albert Street 4, 2100 Godollo, Hungary; 5 Faculty of Health Sciences, Collegium Medicum, Nicolaus Copernicus University, Łukasiewicza 1, 85-821 Bydgoszcz, Poland

**Keywords:** cell culture, cryopreservation, genes, markers, primordial germ cells

## Abstract

Primordial germ cells (PGCs) are the precursors of functional gametes and the only cell type capable of transmitting genetic and epigenetic information from generation to generation. These cells offer valuable starting material for cell-based genetic engineering and genetic preservation, as well as epigenetic studies. While chicken PGCs have demonstrated resilience in maintaining their germness characteristics during both culturing and cryopreservation, their handling remains a complex challenge requiring further refinement. Herein, the study aimed to compare the effects of different conditions (freezing-thawing and in vitro cultivation) on the expression of PGC-specific marker genes. Embryonic blood containing circulating PGCs was isolated from purebred Green-legged Partridgelike chicken embryos at 14–16 Hamburger–Hamilton (HH) embryonic development stage. The blood was pooled separately for males and females following sex determination. The conditions applied to the blood containing PGCs were as follows: (1) fresh isolation; (2) cryopreservation for a short term (2 days); and (3) in vitro culture (3 months) with long-term cryopreservation of purified PGCs (~2 years). To characterize PGCs, RNA isolation was carried out, followed by quantitative reverse transcription polymerase chain reaction (RT-qPCR) to assess the expression levels of specific germ cell markers (*SSEA1*, *CVH*, and *DAZL*), as well as pluripotency markers (*OCT4* and *NANOG*). The investigated genes exhibited consistent expression among PGCs maintained under diverse conditions, with no discernible differences observed between males and females. Notably, the analyzed markers demonstrated higher expression levels in PGCs when subjected to freezing than in their freshly isolated counterparts.

## 1. Introduction

The study of avian primordial germ cells (PGCs) dates back to 1870 when they were first described by Waldeyer. Since then, researchers have focused on understanding the origin, migration, differentiation, and molecular markers of PGCs in birds, notably in species like chicken (*Gallus domesticus*) and Japanese quail (*Coturnix japonica*) [1]. PGCs offer a lot of potential as genetic resources for avian research, especially when studying genetically modified animals [2]. PGCs are the earliest group of germ cells to appear during development and are responsible for generating both oocytes and spermatogonia in adult organisms [1]. These cells are capable of transmitting genetic information to the next generation through gametogenesis [1]. Avian PGCs exhibit distinctive developmental features, such as their unique circulation within the embryonic bloodstream (13 HH–17 HH) before ultimately settling in the genital ridges (28 HH–30 HH) [3,4]. Respectively, during these stages, PGCs can be sourced either from the circulating blood (cPGCs) or from the developing gonads (gPGCs). However, the limited number of PGCs that can be obtained from a single embryo presents a challenge for widespread implementation [4]. Several research endeavors have provided insight into the self-renewal capacity of chicken PGCs, which has resulted in the establishment of protocols for maintaining their growth and proliferation in defined in vitro culture systems for extended periods of time while maintaining their germline characteristics [3]. While the existing protocols for cultivating chicken PGCs can be reproducible, their efficacy differs among breeds, and they are unable to sustain PGCs derived from avian species other than chickens [3,5,6]. A generic protocol remains to be developed for all avian PGCs. Cultivation of PGCs not only makes them readily available in laboratory settings but also allows their use as carriers in transgenic bioreactors and provides a valuable model for studying transgenic chickens [7,8]. Because PGCs allow for the acquisition of the full genetic makeup of the stock, the advent of technologies to manipulate PGCs has provided insights into ex situ conservation [9]. The development of long-term culture systems for chicken PGCs has offered the chance to greatly increase the number of PGCs before cryopreservation and storage for future use [10]. Cryopreservation of PGCs provides support for commercially or industrially important poultry lines or breeds that have undergone extensive selection, serving as a backup in the event of their loss due to pathogen outbreaks, genetic issues, breeding cessation, or natural disasters [9].

The successful development of in vitro cultivation and cryopreservation techniques relies on the acquisition of pluripotency and germline characteristics of PGCs, which in turn are essential for the success of future applications. Various methods of cryopreservation of stem cells across a range of species have been conducted so far (Table 1). PGCs are distinguished by the expression of specific markers that distinctly identify their germ cell lineage apart from somatic cells. Stage-specific embryonic antigen-1 (SSEA-1), a well-established cell surface glycoprotein antigen, serves as a valuable marker for identifying and isolating PGCs within avian embryos [11,12]. This marker is intertwined with the essential roles of PGCs, including cell adhesion, migration, and differentiation [13]. Chicken VASA homologue (CVH) and deleted in azoospermia-like (DAZL), both conserved RNA-binding proteins, exhibit targeted expression exclusively within germ cells throughout germline development [3,14]. Numerous studies have highlighted the pivotal role played by these markers in germline commitment and the intricate process of gametogenesis in invertebrates [3,15,16]. These RNA-binding proteins are essential for sustaining germ cell survival, migration, proliferation, and differentiation [17,18,19,20]. Furthermore, PGCs express several pluripotency-related core transcription factors such as nanog homeobox (NANOG), octamer-binding transcription factor 4 (OCT4), and SOX2, whose expression controls transcription of germness-related genes in these cells [21]. These transcription factors exert precise control over the fate of cells by inhibiting differentiation, thus preserving the cells’ stem cell properties. PGCs lacking these transcription factors may undergo programmed cell death [22] or exhibit compromised migratory capacity, rendering them unable to successfully establish colonies within the gonadal regions after being reintroduced into the embryo’s bloodstream [23]. Studying germ cell-specific genes in depth can reveal their functions in germ cell development and survival, advancing the potential for generating PGC-like cells and in vitro gamete production [24].

Previous studies revealed that PGCs cultured for shorter durations demonstrated better germline competence [6,25]. Hence, cryopreservation of PGCs may also influence their competency, necessitating further analysis of how freezing and thawing cultures may affect PGCs. To our knowledge, the differences in gene expression of germline and pluripotency markers between cryopreserved chicken PGCs and freshly isolated PGCs have not been illustrated. Additionally, no studies have investigated the differences in the impact of short-term and long-term cryopreservation on chicken PGCs. The current study was conducted on the Green-legged Partridgelike chicken, a native Polish breed that demonstrates remarkable adaptability to adverse environmental conditions and exhibits heightened disease resistance compared to other breeds [26]. We aimed in this study to examine how various conditions, namely in vitro cultivation, freezing-thawing, and length of freezing period, affect the expression of marker genes specific to PGCs in Green-legged Partridgelike chickens.

**Table 1:** Overview of cell cryopreservation success by species.

Species	Cell Type	Method(s) of Cryopreservation	Main Cryopreservation Success Indicators	Reference
Chicken	Primordial germ cells	Slow freezing	Gonadal colonization and sperm differentiation post-transplantation	[27]
Drosophila	Primordial germ cells	vitrification	Production of donor-derived gametes	[28]
Rats	Spermatogonial stem cells	Slow freezing	Production of all germ cell types after long-term cryopreservation	[29]
Fish	Germline stem cells	slow freezing	Gonadal colonization post-transplantation	[30]
Human	Induced pluripotent stem cells	slow freezing	Retention of pluripotency and differentiation capacity post-cryopreservation	[31]
Chicken	Primordial germ cells	Slow freezing	Successful migration into gonads	[32]
Horse	Spermatogonial stem cells	vitrification/slow-freezing/fast-freezing	Metabolic activity and spermatogonial stem cell’s protein expression comparable to fresh cells	[33]
Chicken	Primordial germ cells	stored at −150 °C (vitrification)	Viable gametes and offspring produced post-transplantation	[34]
Bovine	Spermatogonial stem cells	Slow freezing	Colonization and proliferation in recipient testes post-transplantation	[35]
Human	Embryonic stem cells	vitrification/slow-freezing	Maintenance of pluripotency	[36]
Rhesus macaques	Spermatogonial stem cells	slow freezing	Retention of engraftment potential post-cryopreservation	[37]

## 2. Materials and Methods

### 2.1. Ethical Considerations

All experimental procedures adhered to the guidelines for the care and use of experimental animals of the University of Science and Technology. The experimental protocols were approved by the Local Ethical Committee for Animal Experiments in Bydgoszcz, Poland (Approval No. 15/2022 from 20.04.2022 r.).

### 2.2. Fertilized Eggs and Incubation

Fertilized eggs from Green-legged Partridgelike chickens were purchased from Zofia i Gracjan Skórniccy-Hodowla Kur Zielononóżek (Duszniki, Poland). Eggs were incubated at a temperature of 37.8 °C and a relative humidity of 60% for 60 h to obtain cPGCs from embryos at the 14–16 HH stage. The eggs were periodically tilted at a 45° angle every 120 min during the incubation process.

### 2.3. Derivation of Embryonic Blood Containing cPGCs

**Figure 1:**
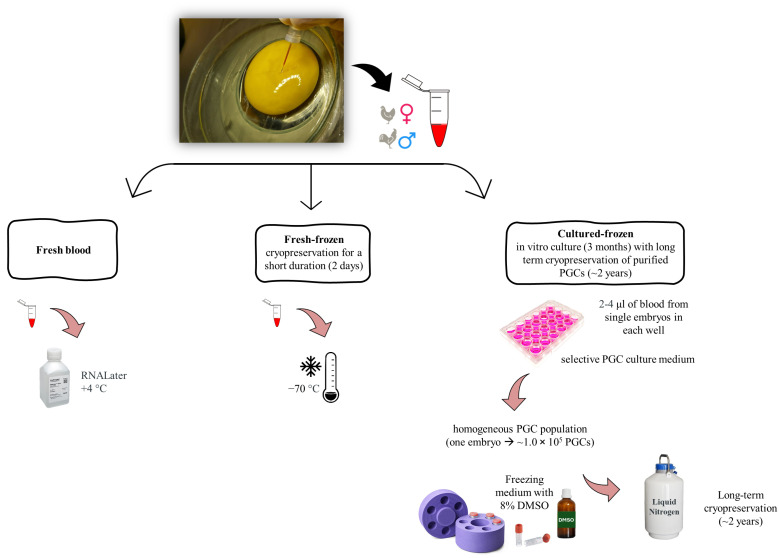
Preparation of samples under three different conditions. PGCs: Primordial germ cells; DMSO: Dimethyl sulfoxide.

Embryonic blood containing cPGCs was isolated from the dorsal aorta of individual embryos under a stereomicroscope using a mouth pipette with fine transfer glass microcapillary of inner diameter 30 µm and outer diameter 40 µm. The isolated blood underwent three different processes (Figure 1): (1) fresh isolation; (2) cryopreservation for a short term (2 days); and (3) in vitro culture (3 months) with long-term cryopreservation of cultured PGCs (2 years). Following isolation, embryos were collected for sex determination and stored at −20 °C until further use.

For freshly isolated blood samples, blood from 20 embryos was placed individually in tubes with RNALater (ThermoFisher, Waltham, MA, USA) and stored at 4 °C until later usage. Once sex determination was done, the samples were pooled into male and female groups. The cells were separated by centrifugation in RNase-free water at 10,000× *g* for three minutes. Subsequently, RNA isolation was carried out using the GeneMATRIX Universal RNA Purification Kit (Eurx, Gdańsk, Poland, cat.no. E3598) following the instructions provided by the manufacturer. For samples cryopreserved for short term, the blood drawn from single embryos was frozen separately as described below. On the other hand, approximately 1–2 μL of blood from single embryos were cultured in vitro in the selective PGC culture medium developed by McGrew and colleagues [38]. The medium consisted of: Calcium-free DMEM (Gibco, Billings, MT, USA, 21068-028), tissue culture-grade water (Gibco, Billings, MT, USA, A12873-01), Sodium Pyruvate (Gibco, Billings, MT, USA, 11360039), MEM vitamin solution (Gibco, Billings, MT, USA, 11120052), MEM amino acids (Sigma, St. Louis, MO, USA, M5550), B27 supplement (Gibco, Billings, MT, USA, 17504044), Glutamax (Gibco, Billings, MT, USA, 35050038), nonessential amino acids (Gibco, Billings, MT, USA, 11140035), nucleosides (EmbryoMax, Munich, Germany, ES-008-D), β-mercaptoethanol (Gibco, Billings, MT, USA, 31350010), CaCl_2_ (Sigma, St. Louis, MO, USA, C4901-100G), ovalbumin (Sigma, St. Louis, MO, USA, A5503), Na heparin (Sigma, St. Louis, MO, USA, H3149-25KU), penicillin–streptomycin mixture (Gibco, Billings, MT, USA, 15070-063), chicken serum (Sigma, St. Louis, MO, USA, C5405), human activin (Invitrogen, Waltham, MA, USA, PHC9564), bFGF2 (Gibco, Billings, MT, USA, 13256-029), and ovotransferrin (Sigma, St. Louis, MO, USA, C7786). While in culture, one-third of the medium was replaced with fresh medium every two days. The cells were cultured for 3 months until a homogeneous PGC population was obtained (Figures S1 and S2). Male and female cell lines were established and then 1.0 × 10^5^ PGCs from each sample were used for long-term cryopreservation. RNA samples were retrieved from resuscitated thawed samples (Figure S3) using the GeneElute Single Cell RNA Purification kit (Sigma-Aldrich, St. Louis, MO, USA, cat.no. RNB300) following the manufacturer’s protocol.

### 2.4. Freezing and Thawing of Cells

**Figure 2:**
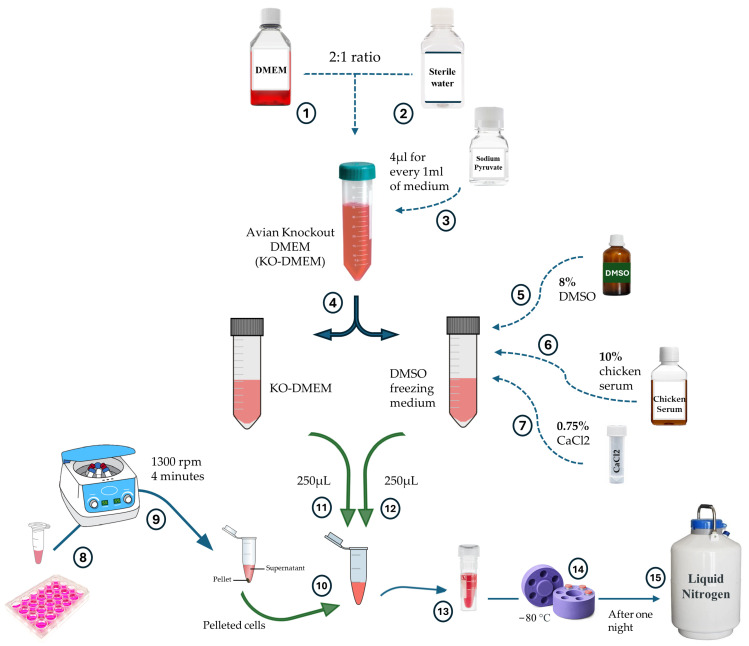
A scheme showing the steps for PGCs cryopreservation. Avian KO-DMEM was prepared by 2:1 ratio of DMEM and sterile water. Then 4 µL of Sodium pyruvate was added to every 1 mL of the medium. The volume of the prepared medium is then divided into two; to one of these parts, DMSO (final concentration 8%), chicken serum (final concentration 10%) and CaCl_2_ (final concentration 0.75%) were added to form the DMSO freezing medium. After pelleting the cells to be frozen and removing the supernatant, the cells were resuspended in 250 µL of avian KO-DMEM. Then, 250 µL of DMSO freezing medium were added slowly. The cell suspension was then transferred to a cryovial which was then placed into −80 °C. For long term storage, the cells were moved to liquid nitrogen after one night.

Freshly prepared freezing media for PGCs was used for freezing both the established PGC lines and the freshly isolated blood. The cryopreservation steps are outlined in Figure 2. The freezing medium was formulated with a 2:1 ratio of DMEM (Thermo Fisher Scientific, Waltham, MA, USA, 21068-028) and sterile water (Thermo Fisher Scientific, Waltham, MA, USA, 15230-089). Additionally, 4 µL sodium pyruvate (Thermo Fisher Scientific, Waltham, MA, USA, 11360-039) was added per 1 mL of medium. To a part of this avian KnockOut DMEM (KO-DMEM) medium, 8% dimethyl sulfoxide (DMSO, Sigma-Aldrich, St. Louis, MO, USA, 276855), 10% chicken serum (Sigma-Aldrich, St. Louis, MO, USA, C5405), and 0.75% 20 mM CaCl_2_ (Sigma-Aldrich, St. Louis, MO, USA, C-34006) were added. The freezing process was done as previously described [32]. Briefly, PGCs containing samples were suspended in 250 µL of DMSO free freezing medium, followed by gentle addition of 250 µL of PGCs freezing medium. The cultured PGCs were kept in nitrogen for up to two years. Fresh blood was kept for two days at −70 °C. For the thawing of PGCs, a solid bead bath at 37 °C was used, and then the total content of the tube was pipetted into 2 mL of culturing media for PGCs. After centrifugation (1000× *g*, 3 min) the supernatant was removed.

### 2.5. Sex Determination

The DNA extraction from each embryo was performed using the QIAamp Fast DNA Tissue Kit (Qiagen, Hilden, Germany, Cat. No. 51404), according to the manufacturer’s instructions. The embryos were homogenized by vortexing with lysis buffer for 30 s followed by incubation in a thermomixer (TS-100C, Biosan, Riga, Latvia) at 1000 rpm for 5 min at 56 °C. The sex of the donor embryos were determined using two pairs of primers: the female-specific *Xhol* W-repeat sequence primer set (5′primer: 5′CCCAAATATAACACGCTTCACT3′; 3′primer: 5′GAAATGAATTATTTTCTGGCGAC3′) and the 18S ribosomal gene sequence (5′primer: 5′AGCTCTTTCTCGATTCCGTG3′; 3′primer: 3′GGGTAGACACAAGCTGAGCC 3′), as described previously by Clinton et al. [39]. The PCR products were separated by electrophoresis, using 2% agarose gel stained with MIDORI Green Advance (NIPPON Genetics, Düren, Germany, cat.no. MG04), at 110 V for 35 min. The DNA bands were then visualized and photographed under G:Box Chemi XR5 (SYNGENE, Cambridge, UK). In female samples, two bands are observed: one corresponding to the female-specific *XhoI* W-repeat sequence with a product size of 415 base pairs, and the other to the 18S ribosomal gene, which is 256 base pairs in size and serves as internal control of PCR. In contrast, male embryos are expected to show only the 18S ribosomal gene sequence (Figure S4).

### 2.6. Quantitative Reverse Transcription PCR (RT-qPCR)

The cDNA was prepared using the smART First strand cDNA Synthesis kit (Eurx, Gdańsk, Poland, cat.no. E0804). The cDNA was amplified by real time qPCR with the primers shown in Table 2. Primers for *SSEA-1*, *CVH* and *DAZL* were designed using Primer3 (v.0.4.1) [40]. The reactions were performed in a 20-µL volume containing 10 ng cDNA; 0.25U UNG (uracil-N-glycosylase); and 15 pmol of each forward and reverse amplification primer in 1× SG qPCR master mix (Eurx, Gdańsk, Poland, E0401). Thermocycling conditions for real time qPCR were as follows: 1 cycle for UNG pre-treatment at 50 °C for 2 min, 1 cycle for initial denaturation at 95 °C for 10 min; and 40 cycles of 94 °C for 15 s, 60 °C for 30 s, and 72 °C for 30 s. Melting-curve profiles were analyzed for all amplicons using the following thermal conditions: 95 °C for 5 s, 70 °C for 1 min, and then a gradual temperature increase to 95 °C at a ramp rate of 0.11 °C/s. Amplification was performed in Roche Light Cycler 480 v. II real-time system (Roche, Basel, Switzerland).

### 2.7. Statistical Analysis

Each sample was measured in triplicate, and fold change gene expression was determined for male and female PGCs in different conditions relative to male fresh-frozen cells, with the male fresh-frozen samples serving as the control/reference (2^−ΔΔCt^ method, where control/reference = 1). All data from RT-qPCR analyses were presented as the mean ± standard deviation (SD) from three independent experiments. GraphPad Prism (version 10.0.1) software (GraphPad Software, La Jolla, CA, USA) was employed for data analysis. Significant differences in relative gene expression were assessed using a two-way ANOVA with Tukey’s multiple comparison test. A *p*-value of less than 0.05 was considered statistically significant.

## 3. Results

**Figure 3:**
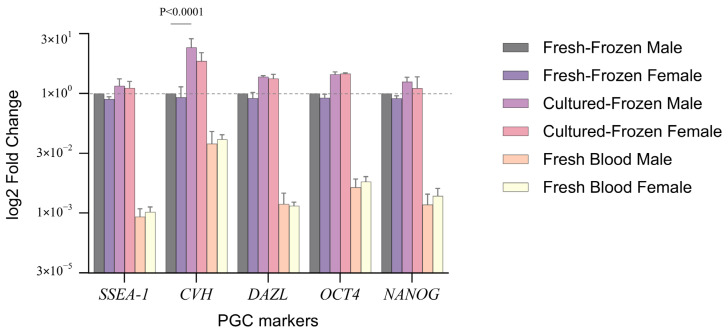
Fold change gene expression was determined for male and female PGC samples in different conditions relative to fresh-frozen male PGCs, with the fresh-frozen male PGC sample serving as the control/reference (2^−ΔΔCt^ method, where control/reference = 1). All data from RT-qPCR analyses were presented as the mean ± standard deviation (SD) from three independent experiments. A *p*-value of less than 0.05 was considered statistically significant. Plotted data are log_2_ transformed.

To investigate the impact of freezing on the expression of the core pluripotent markers and germ-cell specific markers by PGCs, we present Figure 3, which illustrates the relative fold-change of gene expression in male and female PGC samples maintained in the different studied conditions compared to PGCs in fresh-frozen male samples (control). Remarkably, no significant difference in gene expression was observed between male and female samples in all studied conditions. PGCs in female fresh-frozen samples showed consistent expression pattern across all conditions, with no significant deviation from the reference. When comparing PGCs in fresh blood to those in the referenced fresh-frozen samples, it’s observed that PGCs in fresh blood samples generally showed lower expression levels of the studied genes. Cultured-frozen PGCs showed higher expression of the studied genes compared to fresh-frozen cells, but without marked significance, except for the *CVH* gene, which stands out with a significant increase in expression (*p* < 0.0001), particularly in cultured-frozen male PGCs, with a mean equal to 14.5. Overall, fresh-frozen PGCs, frozen for short duration, cultured-frozen PGCs, frozen for long duration, and freshly isolated PGCs showed persistent expression of pluripotency and germline-specific markers. PGCs in fresh blood showed the lowest levels of expression for the studied markers, whereas those cultured-frozen revealed the highest levels of expression.

## 4. Discussion

In this study, we aimed to investigate the impact of different conditions on the expression of pluripotency and PGC-specific marker genes (*SSEA-1*, *NANOG*, *OCT4*, *DAZL*, and *CVH*) in PGCs subjected to either immediate analysis after isolation, cryopreservation for a short term (2 days), or long-term cryopreservation (2 years) after in vitro culturing. We showed that male and female PGCs retained germ cell identity even under conditions of freezing-thawing and in vitro cultivation. No significant differences were observed between the sexes. Furthermore, PGCs subjected to freezing showed higher levels of expression of the aforementioned marker genes than the freshly isolated PGCs.

Altgilbers et al. have examined the expression of PGC-specific genes, including *OCT4*, *NANOG*, *DAZL*, and *CVH*, in both PGCs and chicken embryo fibroblasts [4]. Their findings demonstrated that the pluripotency markers *OCT4* and *NANOG*, along with the specific PGC stem cell markers *SSEA-1*, *DAZL*, and *CVH*, were exclusively expressed in PGCs [4]. In contrast, no expression of these markers was observed in chicken embryo fibroblasts [4]. These results clearly distinguish the gene expression patterns between PGCs and other somatic cells, highlighting the unique expression profiles characteristic of pluripotency and stemness in PGCs. Based on the information available, the expression of the mentioned genes in this study is specifically associated with PGCs found in embryonic blood obtained from 14–16 HH stage embryos.

In line with our study, Tonus et al. have shown that PGC lines, maintained for an extended period in culture (151–540 days), consistently manifested a high proportion of cells expressing SSEA-1 (90–99%), even after cryopreservation [43]. Noteworthy as well, they have unveiled the persistent expression of vital germline-specific markers—*CVH*, *DAZL*, *OCT4*, *NANOG*, *CXCR4*, and other essential genes crucial for effective gametogenesis—across the prolonged cultivation and cryopreservation stages of various cell lines [43]. This cumulative evidence implies the retention of germline competency, thereby maintaining an in vivo-like phenotype.

The higher expression of PGC markers in frozen samples compared to those in unfrozen samples may be attributed to the onset of epigenetic changes, likely caused by DMSO. The cryopreservation of chicken PGCs has been routinely conducted utilizing DMSO as a penetrable cryoprotectant, either individually or in combination with serum as a non-penetrable cryoprotectant, through the method of gradual freezing [27]. The standard method for assessing the effectiveness of cryopreservation is to measure the survival rate of cells after thawing [44]. However, an increasing body of evidence suggests that DMSO may result in alterations to the original epigenetic markers of cells [44]. Although epigenetic mechanisms are pivotal in determining cell fate, there is a limited amount of research available on how various cryobiological factors impact these epigenetic processes. It was demonstrated that in vitro DMSO treatment of mouse embryonic stem cells upregulated pluripotency markers’ mRNA expression [45]. Cryopreserving zebrafish PGCs using cryoprotectants including DMSO, polyvinylpyrrolidone (PVP), and ethylene glycol have been demonstrated to result in downregulation of *CXCR4*, *OCT4*, *VASA*, and *SOX2* transcripts, along with an increase in the expression of heat shock proteins [46]. Notably high levels of DNA methylation were observed only in the promoters of *VASA* (83.6%) and *CXCR4B* (62.1%) [46]. This suggests that DNA methylation may have played a role in reducing the expression of certain genes, like *VASA* and *CXCR4B*. However, for other transcripts like *OCT4* and *SOX2*, reduced transcript levels were not found to be linked to increased promoter methylation [46]. Similarly, another report suggested that cryopreservation with DMSO can reduce the expression of pluripotency markers such as *OCT4* in human embryonic stem cells [47]. However, such changes were not detected in specific types of stem cells, indicating that certain cell types may be less susceptible to the DMSO effect [48].

Research has indicated that DMSO can induce changes in the DNA methylation profile across the genome, particularly at specific gene loci [49]. It was found to induce alterations in the gene expression of DNA methylation enzymes [50]. Existing literature indicates that DMSO can lead to an elevation in the expression of DNA methyltransferases (DNMTs) [49,50]. Following DMSO treatment of cardiac human microtissues, *DNMT1*, a key factor for maintenance of DNA methylation, and *DNMT3A*, which facilitates both de novo and maintenance of DNA methylation, were found to be upregulated while ten-eleven translocation methylcytosine dioxygenase 1 (*TET1*), which plays a key role in active de-methylation, was found to be downregulated [49]. Interestingly, no significant disruption in DNA methylation was observed when analyzing hepatic pathways. Conversely, when mouse embryonic stem cells and embryoid bodies were subjected to DMSO treatment, it was observed that *DNMT1* and *DNMT3B* expression remained unaffected, whereas the expression of *DNMT3A* increased [50]. DMSO can enhance protein levels and catalytic activity through interactions with enzyme substrates, particularly DNA and S-Adenosyl-l-methionine (AdoMet) [51]. Alternatively, DMSO might serve as a methyl donor, potentially inducing hypermethylation [52].

Different results presented by different studies may indicate species-specific and cell-specific effects of DMSO. Hence, investigating the epigenetic consequences of cryopreservation in different models can contribute to a more comprehensive understanding of the cellular mechanisms that can be induced by DMSO upon cryopreservation. To further support the hypothesis that the observed changes in gene expression stem from epigenetic mechanisms, particularly DNA methylation, it is crucial to conduct quantitative analyses of gene expression levels for pivotal enzymes engaged in epigenetic regulation. Additionally, assessing epigenetic markers, with a focus on DNA methylation patterns and histone modifications at pertinent genomic sites, is essential. Furthermore, employing bisulfite sequencing would offer a comprehensive and quantitative evaluation of DNA methylation across gene loci of interest. Alternatively, methylation arrays could provide a feasible method for high-throughput analysis of the methylation status in these crucial regions.

In this study, we have explored the effects of cryopreservation on the gene expression of Green-legged Partridgelike chicken PGCs. The significance of our findings lies in their contribution to avian germplasm conservation. This is particularly relevant for the Green-legged Partridgelike chicken breed, where maintaining genetic diversity is of utmost importance.

To build upon the current study and fully ascertain the utility of PGCs for cryopreservation, we propose several avenues for future research. Firstly, assessing the post-thaw functionality of PGCs will be critical to ensuring they can differentiate into functional gametes. Secondly, long-term viability studies are necessary to monitor the survival and developmental competence of PGCs over extended periods. Thirdly, a comparative analysis of cryoprotectants will help identify the most effective conditions for PGC preservation. Lastly, an examination of the epigenetic impacts of cryopreservation will provide deeper insights into the cellular changes induced by this process.

## Figures and Tables

**Table 2:** Information about primers used for RT-qPCR.

Gene Abbreviation	Gene Name	Primer Sequences		Amplicon Size (bp)	Source
OCT4	Octamer-binding transcription factor 4	F	TCAATGAGGCAGAGAACACG	144	[41]
		R	TCACACATTTGCGGAAGAAG		
CVH (DDX4-VASA)	Chicken Vasa homologue (DEAD-Box Helicase 4)	F	AAGAGGAGCAGTTGGAGGTC	210	This study
		R	AGTAATGGTGCTGGAGGGTC		
DAZL	Deleted In Azoospermia Like	F	TTCGTCAACAACCTGCCAAG	144	This study
		R	TTCACCTCCTTCACAGTACCA		
NANOG	Nanog Homeobox	F	CAGCAGACCTCTCCTTGACC	149	[42]
		R	AAAAGTGGGGCGGTGAGATG		
SSEA-1	Stage-specific embryonic antigen-1	F	GCCACCTACCTGAAGTTCCT	104	This study
		R	TGCTCATCCCAGAAAGACGT		
GAPDH	Glyceraldehyde-3-Phosphate Dehydrogenase	F	ACACAGAAGACGGTGGATGG	193	[42]
		R	GGCAGGTCAGGTCAACAACA		

## Data Availability

All data generated or analyzed during this study are included in this article and the Supplementary Materials.

## Supplementary Materials

The following supporting information can be downloaded at: https://www.mdpi.com/article/10.3390/genes15050624/s1, Figure S1: Chicken PGCs from a representative culture imaged at (A) seeding day, (B) 20 days of culture, and (C) after 50 days of culturing; Figure S2: A representative image of the PGCs in culture during the purification process; Figure S3: Thawed chicken PGCs after long term cryopreservation; Figure S4: Example of the PCR reactions visualization for sex determination of embryos.
