# Supplementary material for: The Effect of Short- and Long-Term Cryopreservation on Chicken Primordial Germ Cells
Source: Genes (Basel). 2024 May 14;15(5):624. doi: 10.3390/genes15050624 (PMC11121574; doi:10.3390/genes15050624)
Supplement: Supplementary file 1 [file genes-15-00624-s001.zip › genes-2989768-supplementary.pdf]

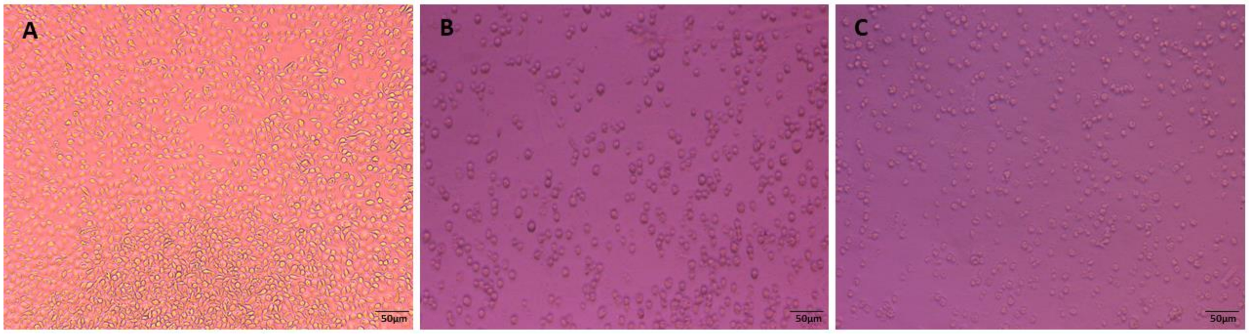

Figure S1: Chicken PGCs from a representative culture imaged at (A) seeding day, (B) 20 days of culture, and (C) after 50 days of culturing. Scale bar: 50 µm.

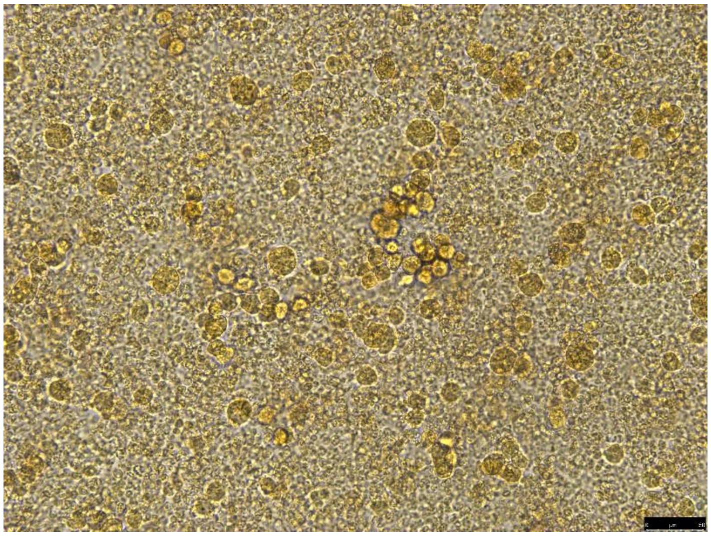

Figure S2: A representative image of the PGCs in culture during the purification process. Scale bar: 50 µm.

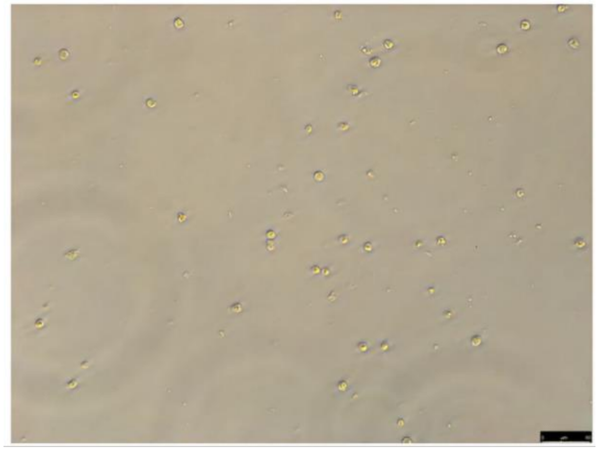

Figure S3: Thawed chicken PGCs after long term cryopreservation. Scale bar: 50µm (Leica DMI8).

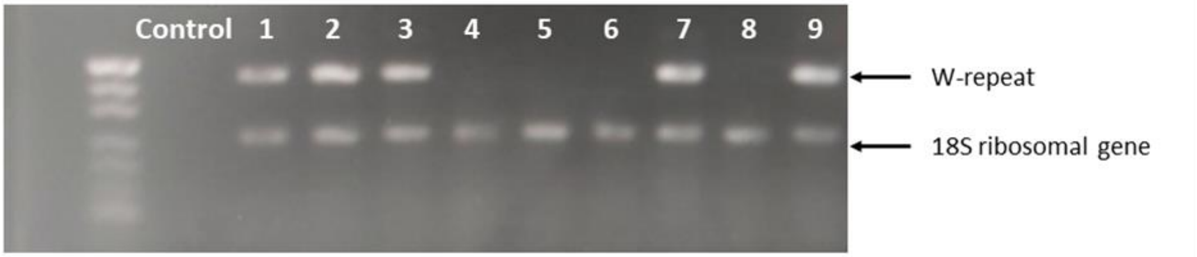

Figure S4: Example of the PCR reactions visualization for sex determination of embryos. In female samples (1, 2, 3, 7 and 9), two bands are observed: one corresponding to the female-specific *XhoI* W-repeat sequence with a product size of 415 base pairs, and the other to the 18S ribosomal gene, which is 256 base pairs in size. In contrast, male embryos (4, 5, 6 and 8) are expected to show only the 18S ribosomal gene sequence.
